# Spectral domain optical coherence tomography guided photodynamic therapy for choroidal hemangioma: a case report

**DOI:** 10.4076/1757-1626-2-8778

**Published:** 2009-09-10

**Authors:** Kakarla V Chalam, Ravi K Murthy, Shailesh K Gupta, Vikram S Brar

**Affiliations:** 1 Department of Ophthalmology, University of Florida, College of Medicine, West 8th Street, Tower II, Jacksonville, 32204, Florida, USA

## Abstract

**Introduction:**

Circumscribed choroidal hemangiomas are vascular tumors associated with secondary changes in the overlying retinal pigment epithelium and neuro-sensory retina. Spectral-domain optical coherence tomography, a recent advancement in fundus imaging techniques provides high resolution images of the retina. We describe spectral domain Optical coherence tomography findings in a case of circumscribed choroidal hemangioma which was successfully treated with photodynamic therapy.

**Case presentation:**

A 41-year-old white male presented with decreased vision in his right eye. Fundus evaluation showed findings consistent with circumscribed choroidal hemangioma. Spectral-domain optical coherence tomography revealed a large serous retinal detachment overlying the tumor with an intact photoreceptor layer. The patient underwent photodynamic therapy and a repeat tomography scan confirmed the resolution of serous detachment with return of normal foveal contour.

**Conclusion:**

Spectral domain optical coherence tomography is an emerging modality in imaging of the retina and reveals ultrastructural changes occurring in various retina pathologies. In this case report we illustrate the use of spectral domain optical coherence tomography for the first time to document retinal changes overlying a choroidal hemangioma and its role as a non-invasive tool in planning the treatment and prognosticating the final visual outcome following treatment for circumscribed subfoveal choroidal hemangiomas.

## Introduction

Choroidal hemangiomas are vascular hamartomas of the choroid and can cause visual loss due to subfoveal location, associated exudative retinal detachment, cystoid macular edema or subretinal fibrosis [1,2]. A number of treatment modalities have been described to treat choroidal hemangiomas including transcleral cryotherapy, radiotherapy, photocoagulation, and in recent times, transpupillary thermotherapy and photodynamic therapy [3,6]-[8].

Spectral domain OCT, in recent times, has been an important advancement in OCT technology, having enhanced speed and sensitivity in acquiring retinal images. It acquires upto 40000 scans in a short period of time and renders a 3-dimensional image with cross-sectional sections providing detailed ultrastructural changes within the retina [4]. In this case report, we document SD OCT changes before and after photodynamic therapy in a case of choroidal hemangioma.

## Case presentation

**Figure 1:**
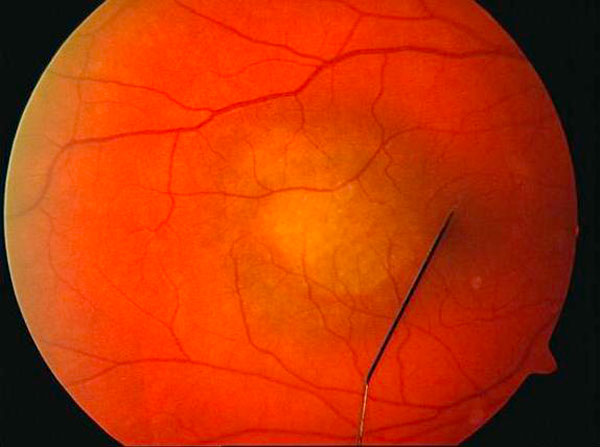
**Color fundus photograph of the right eye of the patient**.

**Figure 2:**
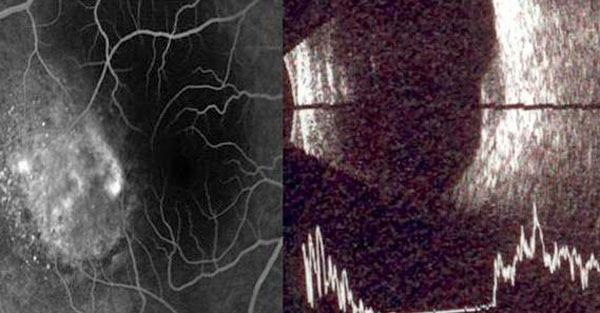
**(a) Fundus fluorescein angiography of the right eye**. **(b)** B-scan Ultra sonogram of the posterior segment.

**Figure 3:**
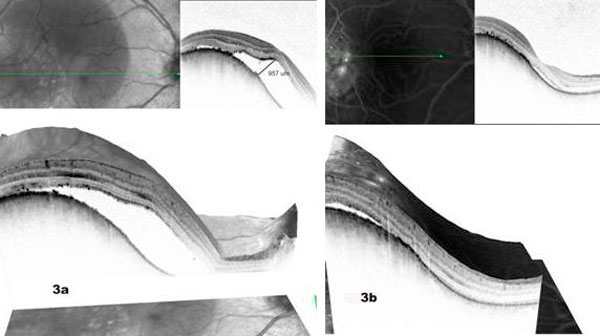
**(a) SD OCT findings of the patient before treatment with photodynamic therapy**. **(b)** SD OCT findings of the patient after treatment with photodynamic therapy.

A 41-year-old white male was referred to our clinic with a diagnosis of right eye submacular edema of one week duration. Systemically there was no associated diabetes mellitus or hypertension. On examination his best corrected visual acuity (BCVA) was 20/50 in the right eye and 20/20 in the left eye. Near vision (J7) and color vision were affected as well in the right eye. Anterior segment examination was unremarkable and dilated fundus evaluation revealed a hypopigmented grey mass that measured 4 disc diameters, located just temporal to the fovea with overlying serous retinal detachment (Figure 1). Fundus fluorescein angiography (Figure 2a) revealed well defined areas of focal hyperfluorescence in the transit phase with late pooling of the dye corresponding to the overlying serous detachment. B scan ultrasonography (Figure 2b) showed a subretinal mass which measured 5.3 mm at the base and 2.64 mm in thickness with corresponding A-scan showing medium to high internal reflectivity. Spectral domain OCT (Spectralis, Heidelberg Engineering Inc) (Figure 3a) revealed a dome shaped elevation of the choroid and focal hyperplasia of the overlying RPE. There was an associated serous retinal detachment overlying the tumor, measuring 957 µm in height at the fovea, but the retinal architecture was preserved with normal photoreceptor layer and absence of intraretinal edema. Systemic evaluation was negative for occult malignancy and patient was diagnosed to have solitary circumscribed choroidal hemangioma (CCH). Photodynamic therapy was applied using a Zeiss laser (Visulas II) emitting a light at 692 nm for photosensitization with prior systemic administration of verteporphin (Visudyne) at a dose of 6 mg/m^2^, with the spot size kept at 6000 microns. The patient was followed up after a week and follow up BCVA was noted to be 20/30. A repeat SD OCT was done which showed minimal residual subretinal fluid, (foveal thickness measuring 269 µm) and return of normal foveal architecture (Figure 3b).

## Discussion

Choroidal hemangiomas are vascular hamartomas which are classified based upon the extent of involvement into two types: Diffuse hemangioma involving much of the choroid in association with Sturge-Weber syndrome and circumscribed involving a portion of the choroid with no associated systemic features [1]. Histo-pathological examinations of the eyes with choroidal hemangiomas enucleated with a mistaken diagnosis of choroidal melanoma have shown that solitary hemangiomas are either cavernous hemangiomas or a mixture of cavernous and capillary hemangiomas. Hyperplasia and fibrous metaplasia of the overlying retinal pigment epithelium are often noted [5].

Clinically on fundus evaluation CCH is typically a solitary dome shaped mass with an orange-red appearance. There can be a grayish appearance due to the fibrous metaplasia of the overlying retinal pigment epithelium [1]. The diagnosis is based upon a strong index of suspicion and presence of findings on A and B scan ultrasonography, which typically show a solid mass with medium to high reflectivity and acoustic solidity. Fibrous and osseous metaplasia of the overlying RPE when present can lead to higher reflectivity [3].

SD OCT is a recent advancement in retinal imaging systems, which uses a non-invasive confocal scanning laser system that transforms a series of optical sections into a layered three-dimensional image of the retina. It provides a detailed image of the changes in the overlying RPE and the neuro-sensory retina and help indirectly in prognosticating the visual potential, following definitive treatment. In our patient, SD OCT showed the preservation of retinal architecture and absence of an intraretinal edema overlying the tumor which helped in good prognostication following effective treatment.

Photodynamic therapy (PDT) has been in recent times found to be an effective treatment for solitary hemangiomas [6]-[8]. PDT allows selective occlusion of the vascular lesions without damaging the adjacent retinal structures. In our case, patient showed symptomatic improvement following PDT which was objectively demonstrated by the reappearance of the normal foveal architecture, resolution of subretinal fluid and disappearance of RPE changes on the SD OCT. Favorable prognostic factors following treatment for subfoveal circumscribed hemangiomas have been described to be good initial visual acuity and duration of the associated neurosensory detachment [2]. SD OCT by demonstrating the changes in the overlying photoreceptor layer can be a useful non invasive tool in prognosticating the final visual outcome following treatment. Documentation of absence of leakage on Indocyaniane Green (ICG) angiography is taken as a direct evidence for regression of the tumor [9]. In our patient, serial SD OCT scans eliminated the need for ICG angiography.

In conclusion, SD OCT is an emerging tool in the imaging of retina and provides high definition ultrastructural changes associated with vitreo-retinal disorders. In this case report we illustrate the use of SD OCT for the first time to document retinal changes overlying a choroidal hemangioma and its role as a non-invasive tool in planning the treatment and prognosticating the final visual outcome following treatment for circumscribed subfoveal choroidal hemangiomas.

## Abbreviations

CCH: circumscribed choroidal hemagioma; ICG: indocyanine green; PDT: photodynamic therapy; RPE: retinal pigment epithelium; SD OCT: spectral domain optical coherence tomography.

## Consent

Written informed consent was obtained from the patient for publication of this case report and any accompanying images. A copy of the written consent is available for review by the Editor-in-Chief of this journal.

## Competing interests

The authors declare that they have no competing interests.

## Authors' contributions

KC and SG identified the case and directly participated in management. They also revised the manuscript and verified its intellectual content. VB and RM worked in collaboration to collect data, acquire clinical photographs, and draft, revise, and reference the manuscript.
